# Pre-operative Carotid Plaque Echolucency Assessment has no Predictive Value for Long-Term Risk of Stroke or Cardiovascular Death in Patients Undergoing Carotid Endarterectomy

**DOI:** 10.1016/j.ejvs.2017.05.013

**Published:** 2017-08

**Authors:** D. de Waard, G.J. de Borst, R. Bulbulia, H. Pan, A. Halliday, ACST-1 collaborative group

**Affiliations:** a Nuffield Department of Surgical Sciences, University of Oxford, Oxford, UK; b Department of Vascular Surgery, University Medical Centre Utrecht, Utrecht, The Netherlands; c Medical Research Council Population Health Research Unit, Clinical Trial Service Unit & Epidemiological Studies Unit, Nuffield Department of Population Health, Oxford, UK

**Keywords:** Carotid artery stenosis, Carotid endarterectomy, Plaque echolucency, Stroke, Randomised controlled trial

## Abstract

**Introduction:**

In patients with carotid stenosis receiving medical treatment, carotid plaque echolucency has been thought to predict risk of future stroke and of other cardiovascular events. This study evaluated the prognostic value of pre-operative plaque echolucency for future stroke and cardiovascular death in patients undergoing carotid endarterectomy in the first Asymptomatic Carotid Surgery Trial (ACST-1).

**Methods:**

In ACST-1, 1832/3120 patients underwent carotid endarterectomy (CEA), of whom 894 had visual echolucency assessment according to the Gray-Weale classification. During follow-up patients were monitored both for peri-procedural (i.e. within 30 days) death, stroke, or MI, and for long-term risk of stroke or cardiovascular death. Unconditional maximum likelihood estimation was used to calculate odds ratios of peri-procedural risk and Kaplan-Meier statistics with log-rank test were used to compare cumulative long-term risks.

**Results:**

Of 894 operated patients in whom echolucency was assessed, 458 plaques (51%) were rated as echolucent and peri-procedural risk of death/stroke/MI in these patients was non-significantly higher when compared with patients with non-echolucent plaques (OR 1.48 [95% CI 0.76–2.88], *p* = .241). No differences were found in the 10 year risk of any stroke (30/447 [11.6%] vs. 29/433 [11.0%], *p* = .900) or cardiovascular (non-stroke) death (85/447 [27.9%] vs. 93/433 [32.1%], *p* = .301).

**Conclusion:**

In ACST-1, carotid plaque echolucency assessment in patients undergoing CEA offered no predictive value with regard to peri-operative or long-term stroke risk or of cardiovascular (non-stroke) death.

**What this paper adds**

In patients with asymptomatic carotid stenosis, plaque echolucency has been shown to predict the risk of ipsilateral stroke and could therefore aid patient selection for preventive surgery. However, carotid plaque echolucency is also thought to predict other cardiovascular events and the value of pre-operative echolucency assessment for post-operative cardiovascular risk is largely unknown. Long-term outcomes of patients undergoing CEA in the ACST-1 trial were assessed with respect to plaque echolucency in the randomised artery at baseline. No differences in risk of stroke or cardiovascular death were found between patients with echolucent and non-echolucent plaques.

## Introduction

Ischaemic stroke and myocardial infarction (MI) are commonly caused by rupture of atherosclerotic plaques and this risk may be related to local plaque instability rather than to the extent of stenotic disease.^1^, ^2^ Several plaque characteristics have been shown to make carotid plaques more prone to rupture and these have been thought helpful in identifying patients at high risk of stroke. Previous studies have shown that carotid plaques with a lipid rich core, intra-plaque haemorrhage, and a thin fibrous cap are positively associated with a past history of cerebrovascular events.^3^, ^4^, ^5^, ^6^ Lipid rich cores appear echolucent on B-mode duplex ultrasound (DUS) assessment, while plaques with “less risky” high fibrous content or calcification appear echogenic.

In patients with asymptomatic carotid stenosis not undergoing carotid revascularisation, carotid plaque echolucency has been associated with a higher risk of future stroke^7^, ^8^, ^9^, ^10^ and it has been suggested as a tool to help aid patient selection for prophylactic carotid endarterectomy (CEA).

Atherosclerosis is a systemic disease and carotid plaque echolucency may reflect plaque instability in other vascular beds as well. Carotid plaque echolucency has been associated with a higher risk of coronary events, even when patients received adequate lipid lowering therapy.^11^, ^12^, ^13^ In a prospective study of 338 endarterectomies, the impact of carotid plaque echogenicity on restenosis, future cardiovascular events, and overall survival was studied. Echolucent carotid plaques (Gray-Weale type I or II) were associated with a significantly higher risk of carotid restenosis and a higher rate of cardiovascular events. However, no difference in overall survival was found, and the prognostic significance of pre-operative echolucency assessment with regard to cardiovascular risk remains largely unknown.^14^

The Asymptomatic Carotid Surgery Trial (ACST-1), the largest randomised controlled trial comparing CEA plus medical therapy versus medical therapy alone in patients with asymptomatic carotid stenosis, has uniquely long, reliable follow-up of both patient cohorts. The present study aimed to assess whether a positive pre-operative carotid plaque echolucency assessment would predict future cardio- and cerebrovascular risk in patients undergoing CEA in ACST-1.

## Methods

### Study design and patient selection

The trial protocol of ACST-1 has been published previously.^15^ Patients were eligible for ACST-1 if they had tight unilateral or bilateral carotid stenosis and no ipsilateral neurological symptoms in the past 6 months. Patients were expected to be available for long-term follow-up. Between 1993 and 2003 a total of 3120 patients were randomised to either immediate CEA or deferral of surgery until it was considered necessary. Both groups received appropriate preventive cardiovascular medical therapy (antithrombotic, antihypertensive and lipid lowering therapy).

The present report includes all patients treated with CEA during the study period, regardless of their initial treatment allocation, and compares those with a randomisation assessment of echolucent versus non-echolucent plaque.

### Plaque echolucency

The grade of stenosis of both carotid arteries was measured with DUS according to local centre protocol and participating centres were asked to assess plaque echolucency of the ipsilateral carotid artery. Plaques were considered to be definitely echolucent when >25% of carotid plaque content was soft (Gray-Weale type 1 or 2) and non-echolucent if soft plaque was uncommon (<25%) or absent (Gray-Weale type 3 or 4).^16^

### Outcome events

The main trial outcomes of ACST-1 were peri-operative mortality and morbidity (stroke and myocardial infarction) and the incidence of non-peri-operative stroke (particularly in the carotid territory of the brain). An independent endpoint review committee, blinded for treatment allocation, adjudicated all major events and further classified strokes wherever possible. Cause specific mortality was ascertained for those participants who died during follow-up.

In the present study, primary endpoints were any stroke occurring after the procedural period (>30 days) and, separately, vascular (non-stroke) death. The secondary endpoint was peri-procedural stroke, MI, and death.

### Statistical analysis

Baseline characteristics of patients with echolucent and non-echolucent plaques were compared using chi-square statistics. A separate analysis of baseline characteristics was performed comparing patients in whom echolucency was assessed with those in whom it was not assessed. For the analysis of non-peri-procedural stroke, patients were censored after their first stroke (i.e. subsequent strokes were not counted). For the analyses of vascular death, all previous events (i.e. non-fatal strokes) were ignored. Kaplan-Meier survival statistics were used to calculate the cumulative risk of primary endpoints and a *p* value was calculated using a log-rank test (pooled over strata). Analysis of peri-procedural events was limited to a patient's first CEA. Unconditional maximum likelihood estimation was used to calculate odds ratios with confidence intervals for the occurrence of peri-procedural events. All analyses were also separately performed for patients allocated immediate CEA. A *p* value of <.05 was considered to be statistically significant for all analyses.

## Results

### Study population

CEA was performed on a total of 1832/3120 (59%) participants. The majority of those allocated immediate CEA had this surgery (1425/1,560, 91%) and usually did so within 1 month (median 27 days). Of those allocated deferral, a total of 407/1560 (26%) underwent surgery over the next decade. Median follow-up after surgery was 75 months for patients allocated to CEA and 45 months for the deferred cohort. Echolucency was assessed in 894/1832 (49%) and in just over half of those a substantial part of the plaque (>25%) appeared echolucent on ultrasound (458/894, 51%). Baseline patient characteristics are summarized in Table 1. Patients with echolucent plaques were slightly younger (*p* = .043) and were more often male (*p* ≤ .001) than patients with non-echolucent plaques. Patients with echolucency assessment had a somewhat tighter ipsilateral stenosis and were more often treated with antihypertensive and lipid lowering therapy at trial entry.

**Table 1:** Baseline characteristics and plaque echolucency.

	Non-echolucent (*n* = 436)	Echolucent (*n* = 458)	*p* value^a^	Not assessed (*n* = 938)	*p* value^b^
Sex (%)					
Male	271 (62)	339 (74)	**<.001**	604 (64)	.082
Age (%), years					
<65	133 (31)	176 (38)	**.043**	267 (29)	**<.001**
65–74	233 (54)	214 (47)		465 (50)	
>75	70 (16)	68 (15)		206 (22)	
Ipsilateral carotid diameter stenosis (%)					
<80	203 (47)	199 (43)	.059	326 (35)	**<.001**
80–89	146 (34)	137 (30)		240 (26)	
90–99	87 (20)	122 (27)		372 (40)	
Contralateral carotid diameter stenosis (%)					
0–49	243 (56)	284 (62)	.087	567 (60)	.232
50–69	96 (22)	75 (16)		196 (21)	
70–99	55 (13)	48 (11)		101 (11)	
Occluded	42 (10)	51 (11)		74 (8)	
Medical history (%)					
IHD	134 (31)	163 (36)	.123	316 (34)	.832
Diabetes	94 (22)	104 (23)	.680	184 (20)	.182
Systolic blood pressure (%)					
>160 mmHg	172 (39)	179 (39)	.911	441 (47)	**.001**
Diastolic blood pressure (%)					
>90 mmHg	153 (35)	169 (37)	.574	369 (39)	.143
Medical therapy at randomisation (%)					
Antiplatelet	392 (90)	401 (88)	.266	840 (90)	.559
Anticoagulant	21 (5)	33 (7)	.134	46 (5)	.285
AHT	279 (64)	282 (62)	.455	636 (68)	**.023**
Lipid lowering	125 (29)	132 (29)	.960	349 (37)	**<.001**
Infarction on imaging (%)					
No	221 (51)	231 (50)	.989	341 (36)	**<.001**
Yes	83 (19)	89 (19)		182 (19)	
Not available	132 (30)	138 (30)		415 (44)	
Previous symptoms either side (%)					
None	278 (64)	279 (61)	.380	557 (59)	.200
>6 months previously	158 (36)	179 (39)		381 (41)	

AHT = antihypertensive; IHD = ischaemic heart disease.

Significant values are in bold (<0.05)

a *p* value between patients with echolucent and non-echolucent plaques.

b *p* value between patients with echolucency assessed and patients with echolucency not assessed.

### Peri-procedural risk

The risk of peri-procedural events according to echolucency status is shown in Table 2. Twenty patients had a fatal peri-procedural event and were excluded from subsequent analyses of long-term risks. Most fatal peri-procedural events were stroke related (13/20, 65%), five were cardiac deaths, and two other causes of death occurred. There were more peri-procedural events in the echolucent group (23/458 [5.0%] vs. 15/436 [3.4%], *p* = .241). This numerical difference was chiefly driven by an excess of fatal events (11/458 [2.4%] echolucent vs. 3/436 [0.7%] non-echolucent, *p* = .039), while strokes in the first 30 post-operative days were similar (19/458 [4.1%] echolucent vs. 12/436 [2.8%] non-echolucent, *p* = .257) as was the 30 day MI rate (4/458 [0.9%] echolucent vs. 3/436 [0.7%] non-echolucent, *p* = .754). The risk of peri-procedural events in the cohort allocated immediate CEA was similar when analysed separately (death/stroke/MI: 16/356 [4.5%] echolucent vs. 13/336 [3.9%] non-echolucent, *p* = .682). The risk of a peri-procedural fatal event was non-significantly higher in this cohort (death: 10/356 [2.8%] echolucent vs. 3/336 [0.9%] non-echolucent, *p* = .064).

**Table 2:** Peri-procedural (<30 days) risk of death/stroke/MI, death/stroke, and stroke.

	Peri-operative death/stroke/MI		
	Observed risk *n* event/*n* patients (%)	OR (95% CI)	*p* value
Non-echolucent plaques (*n* = 436)	15/436 (3.4%)	reference	reference
Echolucent plaques (*n* = 458)	23/458 (5.0%)	1.48 (0.76–2.88)	.241
Echolucency not assessed (*n* = 938)	33/938 (3.5%)	1.02 (0.55–1.90)	.942

	Peri-operative death/stroke		
	Observed risk *n* event/*n* patients (%)	OR (95% CI)	*p* value
Non-echolucent plaques (*n* = 436)	13/436 (3.0%)	reference	reference
Echolucent plaques (*n* = 458)	21/458 (4.6%)	1.56 (0.77–3.16)	.210
Echolucency not assessed (*n* = 938)	26/938 (2.8%)	0.93 (0.47–1.82)	.827

	Peri-operative stroke		
	Observed risk *n* event/*n* patients (%)	OR (95% CI)	*p* value
Non-echolucent plaques (*n* = 436)	12/436 (2.8%)	reference	reference
Echolucent plaques (*n* = 458)	19/458 (4.1%)	1.53 (0.73–3.19)	.254
Echolucency not assessed (*n* = 938)	22/938 (2.3%)	0.85 (0.42–1.73)	.651

	Peri-operative MI		
	Observed risk *n* event/*n* patients (%)	OR (95% CI)	*p* value
Non-echolucent plaques (*n* = 436)	3/436 (0.7%)	reference	reference
Echolucent plaques (*n* = 458)	4/458 (0.9%)	1.27 (0.28–5.71)	.753
Echolucency not assessed (*n* = 938)	9/938 (1.0%)	1.40 (0.38–5.19)	.615

	Peri-operative death		
	Observed risk *n* event/*n* patients (%)	OR (95% CI)	*p* value
Non-echolucent plaques (*n* = 436)	3/436 (0.7%)	reference	reference
Echolucent plaques (*n* = 458)	11/458 (2.4%)	3.55 (0.98–12.82)	.039
Echolucency not assessed (*n* = 938)	6/938 (0.6%)	0.93 (0.23–3.73)	.918

Odds ratio calculated using unconditional maximum likelihood estimation and it's CI, using normal approximation. *p* values are calculated based on chi-square test.

### Long-term risks

#### Risk of stroke

During long-term follow-up beyond 30 days, 59/880 (6.7%) patients suffered a stroke and half of those (30/59, 51%) occurred during the first 5 years. Half could be classified as ischaemic (32/59, 54%) and 5/59 (8%) were haemorrhagic, but the nature of stroke was uncertain in 22/59 (37%) of cases. Ipsilateral ischaemic strokes were uncommon following CEA and were found in 12 patients (12/880, 1.4%) (Table 3). No differences in number of strokes at either 5 or 10 year follow-up between patients with baseline echolucent or non-echolucent plaques were observed. The cumulative 10 year risk of any stroke for echolucent plaques was 11.6% (95% CI 9.4–13.8) vs. 11.0% (95% CI 8.9–13.1) for non-echolucent plaques (*p* = .90). Cumulative 5 year risk of any stroke in patients allocated immediate CEA were doubled for echolucent plaques when compared with non-echolucent plaques in this cohort (12/346 [3.9%] echolucent vs. 6/333 [1.9%] non-echolucent, *p* = .183). However, this non-significant difference disappeared at 10 years (24/346 [11.4%] echolucent vs. 19/333 [9.6%] non-echolucent, *p* = .596).

**Table 3:** Cumulative 5 and 10 year risk of non-peri-procedural stroke by plaque echolucency.

	Any non-peri-procedural stroke					
	Strokes (*n*)	% cumulative 5 year risk (95% CI)	*p* value^a^	Strokes (*n*)	% cumulative 10 year risk (95% CI)	*p* value^a^
Echolucent (*n* = 447)	16	4.2% (3.2–5.2)	.793	30	11.6% (9.4–13.8)	.900
Non-echolucent (*n* = 433)	14	3.7% (2.7–4.7)		29	11% (8.9–13.1)	
Not assessed (*n* = 932)	49	6.2% (5.3–7.1)		65	11.2% (9.7–12.7)	

	Ischaemic non-peri-procedural stroke					
	Strokes (*n*)	% cumulative 5 year risk (95% CI)	*p* value^a^	Strokes (*n*)	% cumulative 10 year risk (95% CI)	*p* value^a^
Echolucent (*n* = 447)	11	3.0% (2.1–3.9)	.725	15	5.4% (3.9–6.9)	.594
Non-echolucent (*n* = 433)	9	2.3% (1.5–3.1)		17	6.7% (4.9–8.5)	
Not assessed (*n* = 932)	34	4.4% (3.7–5.1)		42	6.9% (5.7–8.1)	

	Ipsilateral ischaemic non-peri-procedural stroke					
	Strokes (*n*)	% cumulative 5 year risk (95% CI)	*p* value^a^	Strokes (*n*)	% cumulative 10 year risk (95% CI)	*p* value^a^
Echolucent (*n* = 447)	5	1.4% (0.8–2.0)	.282	7	2.7% (1.6–3.8)	.653
Non-echolucent (*n* = 433)	2	0.5% (0.1–0.9)		5	2.5% (1.2–3.8)	
Not assessed (*n* = 932)	13	1.7% (1.2–2.2)		17	2.9% (2.1–3.7)	

a *p* value is derived by pairwise comparison of echolucent and non-echolucent plaques in log-rank test.

#### Cardiovascular risk

Results for cause specific mortality are shown in Table 4. A total of 341/880 (39%) patients with echolucency assessed died during follow-up, 200 (59%) were caused by vascular disease (including stroke). Cumulative 10 year risk of any vascular death was similar between groups (99/447 [32.5%] echolucent vs. 101/433 [34.4%] non-echolucent, *p* = .542). Analysis of vascular death resulting from causes other than stroke (i.e. mainly cardiac) showed no difference between groups (85/447 [27.9%] echolucent vs. 93/433 [32.1%] non-echolucent, *p* = .301). Similar results were found when patients allocated immediate CEA were analysed separately (non-stroke vascular death: 75/346 [29.4%] echolucent versus 76/333 [31.5%] non-echolucent, *p* = .604) (see Table 5).

**Table 4:** Cause specific numbers of death within 10 years in 880 patients with echolucency assessed and 932 patients in which echolucency was not assessed.

Outcome events		
	EL assessed (*n* = 880)	EL not assessed (*n* = 932)
Cause of death	Number (%)	Number (%)
Stroke	22 (6%)	27 (9%)
Other vascular or cardiac	178 (52%)	135 (45%)
Cancer	67 (20%)	54 (18%)
Respiratory	17 (5%)	20 (7%)
Other known cause	31 (9%)	26 (9%)
Unknown cause	26 (8%)	35 (12%)
Total	341	297

Percentages of total deaths in groups.

**Table 5:** Cumulative 5 and 10 year risk of vascular death by plaque echolucency.

	Any vascular death					
	Deaths (*n*)	% cumulative 5 year risk (95% CI)	*p* value	Deaths (*n*)	% cumulative 10 year risk (95% CI)	*p* value
Echolucent (*n* = 447)	57	14.5% (12.7–16.3)	.747	99	32.5% (29.6–35.4)	.542
Non-echolucent (*n* = 433)	58	15.4% (13.5–17.3)		101	34.4% (31.3–37.5)	
Not assessed (*n* = 932)	109	13.5% (12.3–14.8)		162	27.2% (25.1–29.3)	

	Non-stroke vascular death					
	Deaths (*n*)	% cumulative 5 year risk (95% CI)	*p* value	Deaths (*n*)	% cumulative 10 year risk (95% CI)	*p* value
Echolucent (*n* = 447)	52	13.3% (11.6–15.0)	.609	85	27.9% (25.1–30.7)	.301
Non-echolucent (*n* = 433)	55	14.7% (12.9–16.5)		93	32.1% (29.0–35.2)	
Not assessed (*n* = 932)	93	11.6% (10.5–12.7)		135	22.7% (20.7–24.7)	

*p* value is derived by pairwise comparison of echolucent and non-echolucent plaques in log-rank test.

## Discussion

Plaque echolucency is a non-invasive measurement of plaque “stability” and previous studies have shown it to be associated with an increased risk of stroke^7^, ^8^, ^9^, ^10^, ^17^ and myocardial infarction.^11^, ^12^, ^13^

The present study assessed whether carotid plaque echolucency predicted future stroke or cardiovascular death after resection of the unstable plaque. While it is expected that the risk of ipsilateral stroke after CEA would be similar for both groups, the risk of any stroke or vascular death from causes other than stroke may remain elevated in the echolucent group if this reflects plaque stability of other vascular beds.

The present study confirmed that, following carotid surgery and resection of the plaque, there was no difference in risk of ipsilateral stroke between patients with echolucent or non-echolucent plaques. In contrast, in a previous study of the ACST-1 (deferred) cohort who did not undergo surgery, patients with echolucent plaques had a significantly higher 5 year risk of ipsilateral stroke.^10^

No association was found between plaque echolucency and risk of any stroke or cardiovascular death. This finding contradicts the hypothesis that individuals with local plaque instability have a systemic predisposition to develop unstable plaques in other vascular beds. In a study of 3007 ECST patients with symptomatic carotid stenosis, it was shown that patients with irregular plaques on angiogram of the symptomatic (ipsilateral) carotid artery were likely to also have irregularity on the contralateral side. Moreover, it was shown that patients with irregular plaques in both arteries had the highest risk of previous MI (130/846 [15%] vs. 44/510 [9%]; *p* < .001) and 10 year risk of non-stroke vascular death (45% vs. 14%, *p* < .001) when compared with patients with smooth arteries.^18^ The increasing use of statins in ACST-1 during the long follow-up period may have protected both groups of patients from cardiovascular events, especially MI.

Several factors may explain the lack of association between echolucency and cardiovascular risk. First, atherosclerosis is a chronic disease and the carotid plaque composition may change over time. The present results, based on a single ultrasound scan at time of randomisation, may be influenced by interim changes in the individuals' plaque composition during follow-up. Changes in plaque composition may also have been influenced by a number of factors and improvements in risk factor control and medical therapy could have played an important role.

Statins stabilise carotid and coronary plaque and during ACST-1 statins became widely used in clinical practice. The use of statins in the ACST-1 rose from 33% at randomisation (even lower in the early years) to 39% at 2 year follow-up, 55% at 5 years, and 68% at 10 years. Statin therapy between groups did not differ at baseline, but they were used more often in the echolucent group at 5 years follow-up (52% vs. 42%).

In the METEOR trial it was shown that, in patients with subclinical atherosclerosis, rosuvastatin effectively stopped the progression of carotid atherosclerosis.^19^ The ASTEROID trial, using intravascular ultrasound of the coronary arteries, showed regression of coronary artery atherosclerosis in patients who were taking 40 mg rosuvastatin.^20^ These effects on atherosclerotic disease may later have significant effects on clinical outcome, as shown in several other trials.^21^, ^22^, ^23^

In the large, mostly symptomatic Athero-Express study, carotid plaque histology showed a temporal decrease in features normally associated with plaque instability (large lipid core, high macrophage count, intraplaque haemorrhage). This decrease was associated with improved risk factor control and better medical therapy. However, the change towards more “stable” plaque composition did not lead to a measureable reduction in cardiovascular events during follow-up.^24^

In this ACST-1 study more peri-procedural events were found in patients with echolucent plaques. The risk of peri-procedural death was significantly increased and over three times higher in those with echolucent plaques (11/458 [2.4%] echolucent vs. 3/436 [0.7%] non-echolucent, *p* = .039), but such results based on a small number of procedural events may simply be chance, and these have not been replicated in other studies.

In patients undergoing carotid artery stenting, plaque echolucency is associated with an increased risk of peri-procedural stroke.^25^, ^26^

The present study is one of the first to assess the predictive value of echolucency following resection of the unstable plaque, and one of its strengths is that ACST-1 had long follow-up and well characterized endpoints, making results reliable.

However, there are several limitations. First, assessment of echolucency was only reported for about half of patients included in ACST-1. However, this still represented a large cohort and many participating centres. In ACST-1, centres usually assessed echolucency in all or none of their patients, thereby reducing patient selection bias. Second, plaque echolucency measured by the Gray-Weale classification is (in this and other studies) subjective and somewhat operator dependent. Moreover, the binary nature of the present data excluded potential effects from a more gradual scale of plaque stability.

### Conclusion

In this large trial, baseline carotid plaque echolucency assessment did not predict peri-procedural or long-term stroke risk or (non-stroke) cardiovascular death.
